# Perceived satisfaction, perceived usefulness, and interactive learning environments as predictors of university students’ self-regulation in the context of GenAI-assisted learning: an empirical study in mainland China

**DOI:** 10.3389/fpsyg.2025.1599478

**Published:** 2025-12-03

**Authors:** Zhiwei Liu, Yan Zhao, Haode Zuo, Yongjing Lu

**Affiliations:** 1 School of Mathematics, Yangzhou University, Yangzhou, China; 2 Department of Mathematics, Taizhou University, Taizhou, China

**Keywords:** GenAI-assisted learning, perceived self-regulation, structural equation modeling, three-tier models of self-regulation, prospective mathematics teachers

## Abstract

Given the potential risks of learners’ misuse of generative artificial intelligence (GenAI), including over-reliance, privacy concerns, and exposure to biased outputs, it is essential to investigate university students’ self-regulation in GenAI-assisted learning. Self-regulated learning enables university students to set goals, monitor their learning progress, and adjust strategies, thereby enhancing the effectiveness of GenAI-assisted learning. Guided by the three-tier model of self-regulation, which encompasses individual characteristics, cognitive and emotional factors, and behavioral intention, this study employed a mixed-method approach. Structural equation modeling (SEM) was used to quantitatively examine the relationships among key variables, while interviews provided qualitative insights, enabling a comprehensive exploration of factors influencing self-regulation in GenAI-assisted learning. Using a sample of 607 university students (e.g., prospective mathematics teachers) from Mainland China, this study found that compared to perceived self-efficacy and interactive learning environments, information system quality showed a stronger influence on learners perceived usefulness and satisfaction in GenAI-assisted learning. In predicting learner perceived self-regulation, perceived usefulness was a stronger predictor than the interactive learning environment and perceived satisfaction. Similarly, perceived usefulness was a stronger predictor of behavioral intention than perceived satisfaction and self-regulation. This study further investigated the partial mediating effects of perceived usefulness, perceived satisfaction, and perceived self-regulation among other variables. This study proposes a conceptual model to explore the interconnectedness of these factors in GenAI-assisted learning. It highlights the importance of information system quality for educators and recommends that researchers further investigate the dynamic factors influencing self-regulation in GenAI-assisted learning environments.

## 1 Introduction

GenAI, represented by tools such as OpenAI’s ChatGPT and Baidu’s Ernie Bot, has recently gained significant attention in higher education (Liu et al., 2025a). These tools can assist learners by delivering timely feedback, generating customized learning materials, and supporting technology-enhanced instruction (Zhang and Tur, 2024). With the growing integration of GenAI tools in higher education, their potential to create engaging learning experiences has been widely recognized, for example, by enabling multilingual translation, correcting programming errors, generating narratives, and acknowledging mistakes (Al-Emran et al., 2023; Hwang and Chang, 2023; Jeon, 2024; Pan et al., 2025).

However, the rapid adoption of GenAI has also raised concerns about potential risks, including privacy risks, over-reliance on GenAI outputs, exposure to inaccurate or biased information, and diminished critical thinking (Rospigliosi, 2023). Strengthening learners’ self-regulation is widely recognized as essential for the effective use of GenAI in higher education (Karal and Sarialioglu, 2025). Self-regulation empowers learners to set goals, monitor their progress, and adjust strategies proactively, enabling them to critically evaluate GenAI-generated content, avoid over-reliance on GenAI, and manage potential privacy risks (Sallam et al., 2025). Consequently, enhancing self-regulation can reduce the risks of GenAI misuse and support deeper autonomous learning. Despite the rapid integration of GenAI tools such as OpenAI’s ChatGPT and Baidu’s Ernie Bot into higher education, most existing studies have focused on technology acceptance (Du and Lv, 2024; Biyiri et al., 2024), behavioral intention (Garcia, 2025), and specific applications (e.g., AI-assisted writing and chatbot adoption; Xia et al., 2023; Hu et al., 2025), rather than on learners’ self-regulation learning. Current studies have predominantly adopted Unified Theory of Acceptance and Use of Technology Model (UTAUT), Technology Acceptance Model (TAM), or extended acceptance models, focusing on variables such as perceived usefulness, perceived ease of use, and behavioral intention (Şimşek et al., 2025; Tang et al., 2025; Liu et al., 2025b). However, it has largely overlooked the psychological and contextual mechanisms that shape learners’ self-regulated learning in GenAI-assisted environments, including cognitive strategies, emotional regulation, and learning environment factors. To date, empirical research integrating psychological, emotional, and contextual dimensions are scarce, and very few have used SEM to systematically explain how these factors shape learners’ self-regulation in higher education. This underscores a critical thematic gap, namely the need to develop an integrative understanding of how cognitive, emotional, and contextual factors jointly influence learners’ self-regulation in GenAI-assisted learning. Addressing this gap is essential for promoting the effective use of GenAI tools and guiding the design of pedagogical strategies in the era of GenAI-supported education.

To address this gap, the present study draws upon Liaw and Huang’s (2013, p. 6) three-tier models of self-regulation, which is expanded in this study into eight variables, namely perceived self-efficacy, perceived anxiety, information system quality, interactive learning environment, perceived usefulness, perceived satisfaction, self-regulation, and behavioral intention. Prior studies have consistently identified these variables as key determinants of learners’ self-regulation in educational settings (Bandura, 1986; Davis, 1989; DeLone and McLean, 1992; Liaw and Huang, 2007; Sun et al., 2008). These variables collectively capture the cognitive, emotional, and contextual dimensions essential for understanding how learners interact with GenAI and regulate their learning behaviors.

## 2 Literature review

### 2.1 GenAI in higher education

GenAI is transforming higher education by offering students innovative approaches to accessing knowledge and enhancing their learning processes (Strzelecki and ElArabawy, 2024). GenAI, such as OpenAI’s ChatGPT and Baidu’s Ernie Bot, have transformed the learning process by providing personalized support, thereby enhancing students’ learning performance (Wang et al., 2024). These tools foster deeper cognitive processing and enhance students’ interest in problems-solving (Jin et al., 2025a). For instance, these tools can generate case studies, role-playing scenarios, and debate topics, which stimulate students’ ideas (Chan and Hu, 2023; Lodge et al., 2023). By using GenAI, students can enhance their ability to organize learning tasks, monitor progress, and receive immediate feedback, all of which are essential components of self-regulated learning. Furthermore, GenAI can provide customized learning paths to enable students to engage in adaptive learning experiences (Yu et al., 2025). This contributes to improved time management, higher engagement, and increased motivation, as learners gain a greater sense of control over their learning processes. Recognizing these benefits, educators have increasingly integrated GenAI tools to foster dynamic learning environments that promote active learning, and critical thinking (Tang et al., 2025).

In the context of Mainland China, the rapid integration of GenAI into higher education have fostered a distinctive environment for student learning. GenAI, such as OpenAI’s ChatGPT and domestic platforms like Baidu’s ERNIE Bot, are being increasingly adopted in Chinese universities, providing functions such as intelligent question-and-answer (Q&A) systems, academic writing support, and content generation. Surveys data indicate that GenAI-assisted functions, including paper editing and Q&A systems, have reached a penetration rate of approximately 84% in Chinese universities (Zhang and Wang, 2025). This widespread adoption reflects China’s policy emphasis on advancing educational digitalization and enhancing university students’ digital literacy. However, Chinese higher education is characterized by unique cultural and institutional features that affect how students engage with GenAI-assisted learning (Huang et al., 2019). For example, Chinese students are typically socialized within teacher-centered and exam-oriented educational environments, which may limit their capacity for self-regulation in interacting with GenAI tools (Yan et al., 2024). Moreover, concerns regarding academic integrity and data privacy are pronounced in Mainland China, where institutional trust and adherence to national AI regulations play a central role in shaping student adoption behavior (Teo and Huang, 2019). Therefore, investigating Chinese university students’ self-regulated learning in GenAI-assisted environments is timely and significant, as it captures an educational ecosystem characterized by distinctive cultural, institutional, and technological dynamics.

However, the integration of OpenAI’s ChatGPT and Baidu’s Ernie Bot into higher education also presents risks and challenges, particularly concerning self-regulated learning (Jin et al., 2025b). A primary concern is the potential overreliance on these tools, which may hinder students’ active engagement in critical thinking and reflection (Strzelecki, 2024). By relying on GenAI for immediate responses, students may bypass essential cognitive processes, which can impair their ability to set learning goals and monitor progress (Zhou et al., 2024). Furthermore, sensitive information disclosed during interactions may be vulnerable to breaches or misuse, potentially eroding students’ confidence in employing these tools (Chiu, 2024). Also, GenAI-generated content may carry biases from its training data, potentially resulting in inaccuracies that distort students’ decision-making (Farrokhnia et al., 2024). This limitation undermines students’ ability to critically evaluate generated content and hinders the development of their self-regulation learning. Concerns about academic integrity also arise because GenAI tools can generate assignments, essays, or creative works, undermine the authenticity of student submissions and hinder the development of independent learning (Bhullar et al., 2024). Finally, university students with limited technical proficiency may encounter technological barriers that hinder their effective use of GenAI, thereby constraining their self-regulation learning (Fergus et al., 2023).

To address these risks and foster their self-regulated learning, universities should focus on cultivating students’ digital literacy, enabling them to use GenAI effectively while retaining autonomy (Wang et al., 2024). Establishing clear ethical standards and safeguarding data privacy are essential for creating a responsible educational environment (Cotton et al., 2024). Ensuring transparency in AI algorithms and content generation further enables students to critically evaluate GenAI outputs and ensures that these tools support their self-regulation learning (Arthur et al., 2025).

### 2.2 Learners’ self-regulation in GenAI-assisted learning based on three-tier models

In education, self-regulation refers to learners’ ability to actively manage their cognitive, emotional, and behavioral processes in the learning process (Kim et al., 2023). It involves setting goals, monitoring progress, adjusting learning strategies, and maintaining motivation. In the context of GenAI-assisted learning, self-regulation is particularly critical. Using GenAI tools such as ChatGPT or Baidu’s ERNIE Bot requires learners to effectively balance tool interaction with the active management of their own learning processes (Wang, 2024). Self-regulation encompasses not only traditional cognitive strategies but also the ability to adapt to the distinctive affordances of GenAI tools (e.g., content generation, feedback loops, and personalized learning; Hew et al., 2025). These tools support learners in organizing their learning process, while they also require students to actively monitor their interactions with the technology to prevent over-reliance (Zhang and Tur, 2024). Therefore, in this study, self-regulation is defined as students’ capacity to manage their learning experiences in GenAI-assisted learning environments by adjusting their strategies, behavior, and emotions to achieve the intended learning outcomes (Xu et al., 2025).

**Figure 1:**
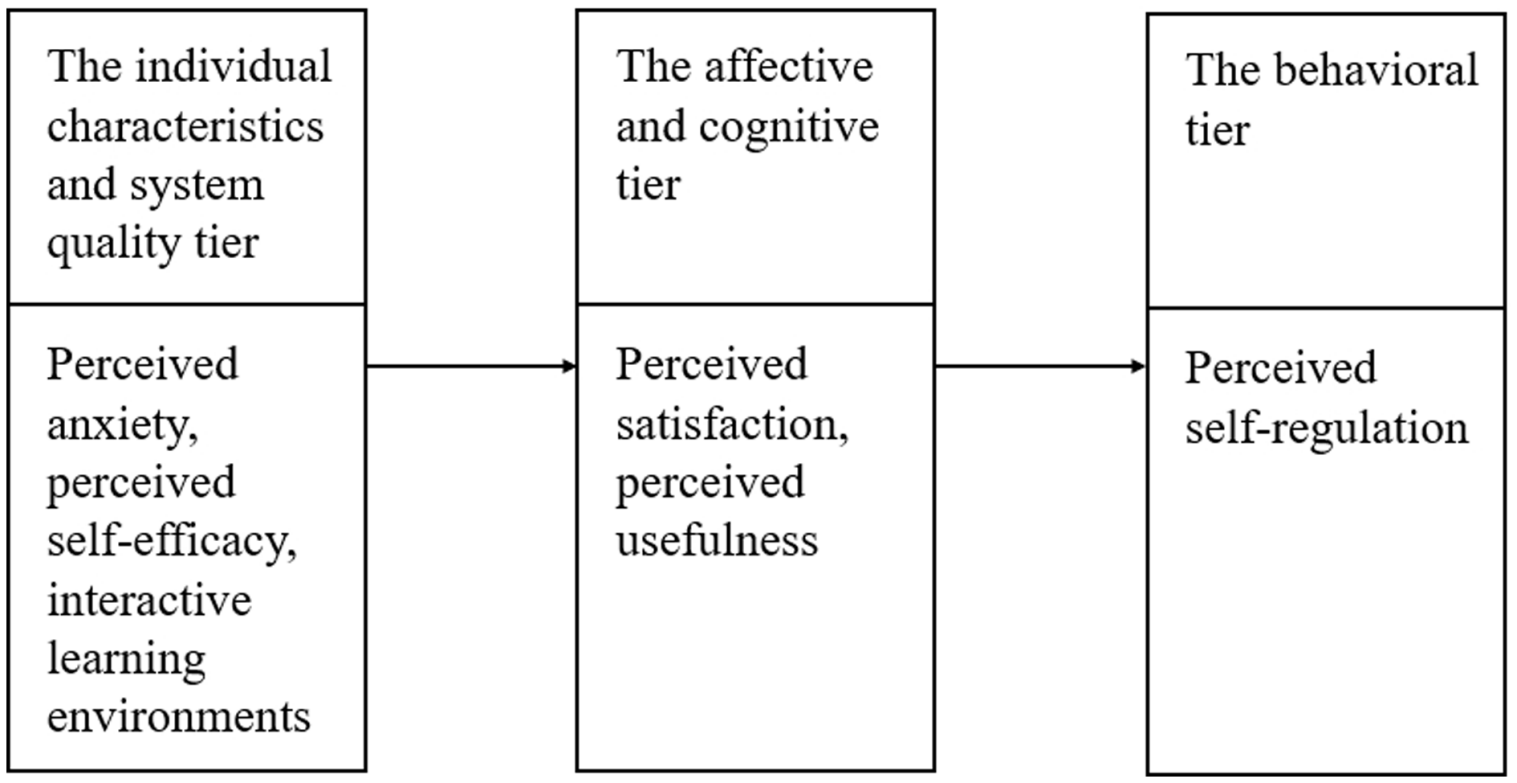
The conceptual model of understanding self-regulation (Liaw and Huang, 2013).

To understand and measure self-regulation in GenAI-assisted learning, Liaw and Huang (2013) proposed a three-tier model, which has become an important framework in e-learning research (Figure 1). This model comprises three interrelated tiers that shape how learners regulate their behaviors in technology-supported learning. The first tier highlights individual characteristics (e.g., self-efficacy and anxiety) and environmental factors (e.g., information system quality), which influence learners’ cognitive and affective responses during the learning process. High-quality GenAI tools (e.g., OpenAI’s ChatGPT, Baidu’s Ernie Bot) can alleviate learners’ cognitive load and offer more personalized learning experiences, thereby fostering their self-regulation learning. The second tier encompasses cognitive and affective factors, including perceived satisfaction and usefulness. These factors shape learners’ perceptions of the tools they use, thereby influencing their cognitive and affective responses. The model posits that learners who perceive tools as satisfying and useful are more likely to engage in self-regulation learning. Finally, the model highlights the behavioral intention tier, which is driven by perceived self-regulation. In this study, this tier reflects the learner’s intention to continue using GenAI, based on their usefulness and satisfaction. In other words, learners who can effectively regulate their learning with GenAI are more likely to sustain their use of these tools in the future.

Although TAM and UTAUT are widely applied to examine technology acceptance, they primarily emphasize perceived ease of use, perceived usefulness, and behavioral intention, focusing on the technological aspects of users’ interactions with tools. However, these models overlook the cognitive, emotional, and behavioral dynamics that are crucial for understanding self-regulated learning in the context of educational technologies (Şimşek et al., 2025; Liu et al., 2025b). In contrast, Liaw and Huang’s (2013) three-tier self-regulation model offers a more comprehensive framework by integrating individual characteristics, emotional responses, and behavioral intention, providing a deeper understanding of how learners interact with GenAI tools such as ChatGPT and Baidu’s ERNIE Bot in higher education. This model extends the TAM/UTAUT frameworks by addressing the psychological and emotional factors that influence self-regulation in technology-mediated learning environments. While TAM and UTAUT primarily examine user acceptance based on technology perceptions, the three-tier model incorporates learners’ self-efficacy, anxiety, and emotional responses, which are particularly important in GenAI-assisted learning. It highlights the role of individual traits and contextual factors in shaping how students engage with GenAI tools, balancing cognitive, emotional, and behavioral aspects to achieve their learning goals. The novelty of applying the three-tier model in the context of GenAI-assisted learning lies in its ability to address the interplay between learners’ personal characteristics, their emotional responses, and the quality of the GenAI tools they use. Unlike the more limited scope of TAM/UTAUT, this model provides a richer, more nuanced understanding of how self-regulation influences learners’ use of GenAI tools. This theoretical framework offers valuable insights into the psychological, emotional, and contextual factors that shape learners’ self-regulation and how they perceive and interact with GenAI tools in their academic learning environments. Consequently, this expanded framework contributes to a deeper theoretical understanding of self-regulated learning in GenAI-enhanced education.

### 2.3 SEM

SEM is a powerful statistical technique for examining complex relationships between observed and latent variables (Anderson and Gerbing, 1988). Unlike traditional regression analysis, SEM enables researchers to test simultaneous causal pathways among multiple variables, including direct and indirect effects (Fan et al., 2016). This is particularly valuable for investigating constructs such as self-regulation, which encompass interrelated cognitive, emotional, and behavioral factors that cannot be directly measured. In this study, SEM was employed to construct a theoretical framework for examining the factors that influence learners’ self-regulation in GenAI-assisted learning environments. By using SEM, this study examined how individual characteristics (e.g., self-efficacy and anxiety), environmental factors (e.g., information system quality), and cognitive and affective responses (e.g., perceived satisfaction and usefulness) interrelate to shape learners’ self-regulation and behavioral intentions. This approach allows for the evaluation of both the direct effects of these factors and the modeling of their indirect effects. The use of SEM in this study is justified by its ability to handle complex data structures, accommodate measurement error, and model latent variables, which are central to this research (Fornell and Larcker, 1981). Unlike other modeling techniques, SEM offers a more nuanced understanding of how multiple factors, including learners’ emotional and cognitive responses, influence their learning behaviors. Given the dynamic and personalized nature of GenAI (e.g., OpenAI’s ChatGPT and Baidu’s Ernie Bot), SEM enables the capture of the complex interactions between learners and these tools, as well as their potential to support or hinder self-regulated learning. Overall, SEM serves as an appropriate and essential methodological approach for exploring the psychological, emotional, and contextual factors that influence self-regulation in GenAI-assisted learning. It provides a holistic understanding of how interactions with GenAI tools influence learners’ behaviors, forming a cornerstone for this study’s design.

### 2.4 Perceived self-efficacy

Bandura (1986) defined perceived self-efficacy as an individual’s confidence in their ability to successfully accomplish a specific task. In this study, perceived self-efficacy refers to learners’ confidence in using GenAI tools to achieve their learning goals (Börekci and Uyangör, 2025). Kumar (2021) reported that the effect of learners’ self-efficacy on chatbot-assisted learning was limited. However, some studies have suggested that higher self-efficacy can enhance learners’ learning performance and persistence, as it is associated with a more positive attitude toward this learning approach (Chang et al., 2022; Divekar et al., 2022). For instance, Lee et al. (2022) found that GenAI not only supported students in understanding knowledge but also offered immediate solutions to the problems they encountered, thereby enhancing their learning confidence. Therefore, perceived self-efficacy is crucial for GenAI-assisted learning.

### 2.5 Perceived anxiety

Computer anxiety is an uncomfortable emotional state characterized by nervousness, worry, and apprehension (Doll and Torkzadeh, 1988). In this study, perceived anxiety refers to an individual’s tendency to feel uneasy or fearful about the current or potential use of GenAI (Sallam et al., 2025). This anxiety may be exacerbated by the need for users to learn new terminology and understand unfamiliar applications associated with GenAI (Caporusso, 2023). Also, issues such as user privacy breaches and virus intrusions increase the risks of internet usage, further intensifying learners’ anxiety (Barbeite and Weiss, 2004). Existing research has indicated a negative correlation between perceived anxiety and the frequency of GenAI use (Zhu et al., 2024). Therefore, perceived anxiety is a critical factor influencing the use of GenAI.

### 2.6 Information system quality

In this study, information system quality is defined as the extent to which the information generated by GenAI accurately conveys the intended meaning (Baabdullah, 2024). It encompasses the technical performance of the system, the quality of the generated content, and the effectiveness of its practical applications. Mun and Hwang (2024) indicated that the information system quality of ChatGPT significantly influenced learners’ intention to use them. Similarly, Tlili et al. (2023) found that the reliability and flexibility of GenAI were crucial for ensuring stable and efficient operation. Furthermore, the content quality of GenAI is reflected in the accuracy and completeness of its generated information, which is intended to provide users with high-quality responses (Goli et al., 2023). For example, a high level of information system quality can effectively encourage learners to engage with live chat features during the learning process (McLean and Osei-Frimpong, 2019). Therefore, information system quality is a crucial determinant of GenAI-assisted learning.

### 2.7 Interactive learning environments

An interactive learning environment refers to a setting that enhances learning experience by facilitating interactions among learners, instructors, peers, and learning systems (Liaw, 2008). GenAI fosters interactive learning by responding to learners’ queries, which lies at the core of interactive engagement (Rospigliosi, 2023). In GenAI-assisted learning, synchronous and asynchronous features create dynamic communication channels. Synchronous communication enables real-time interaction between teachers and students, while asynchronous communication supports flexible participation without simultaneous engagement (Sharma et al., 2007). These interaction modes not only enable learners to share information but also allow them to access valuable knowledge autonomously. Because learning inherently occurs in social contexts, interactions among learners, instructors, and peers offer critical opportunities for knowledge construction (Liaw and Huang, 2013). Consequently, an interactive learning environment is essential for GenAI-assisted learning.

### 2.8 Perceived usefulness

The usefulness of learning systems is recognized as a critical factor influencing learning performance (Virvou and Katsionis, 2008). In this study, perceived usefulness is defined as the extent to which learners perceive GenAI as effective, efficient, and satisfying in supporting their learning (Kim et al., 2025). Prior research has shown that interactive learning environments were key determinants of learners perceived usefulness in e-learning systems (Liaw and Huang, 2007). Börekci and Uyangör (2025) found that perceived self-efficacy significantly impacted perceived usefulness. Zheng W. et al. (2024) and Zheng Y. et al. (2024) also suggested that the information system quality significantly affected perceived usefulness. Furthermore, Sallam et al. (2025) demonstrated a negative correlation between perceived anxiety and perceived usefulness. Based on these findings, we propose the following hypotheses:

*H1*: Perceived self-efficacy has a positive impact on perceived usefulness of learners in GenAI-assisted learning.

*H2*: Perceived anxiety has a negative impact on perceived usefulness of learners in GenAI-assisted learning.

*H3*: Information system quality has a positive impact on perceived usefulness of learners in GenAI-assisted learning.

*H4*: Interactive learning environment has a positive impact on perceived usefulness of learners in GenAI-assisted learning.

### 2.9 Perceived satisfaction

Perceived satisfaction is considered a key indicator of the effectiveness of learning systems (Virvou and Katsionis, 2008). In this study, it is defined as the degree of comfort and contentment learners experience in using GenAI (Kim et al., 2025). Existing studies have shown that perceived self-efficacy, information system quality, interactive learning environments, and perceived usefulness significantly influenced perceived satisfaction (Kim and Ong, 2005; Liaw, 2008). Specifically, Liaw et al. (2007) found that perceived self-efficacy played an important role in shaping learners’ satisfaction with e-learning systems. Liu et al. (2009) reported that learner satisfaction increases when e-learning environments provide more interactive activities. Further, the information system quality is closely associated with learners’ satisfaction (Almufarreh, 2024; Wut and Lee, 2022). Al-Sharafi et al. (2023) also shown that learners are satisfied with GenAI-assisted learning when the tools meet their needs. Moreover, prior studies have identified a negative correlation between perceived anxiety and perceived satisfaction (Sun et al., 2008; Tsai, 2009). Based on these findings, the following hypotheses were formulated:

*H5*: Perceived self-efficacy has a positive impact on perceived satisfaction of learners in GenAI-assisted learning.

*H6*: Perceived anxiety has a negative impact on perceived satisfaction of learners in GenAI-assisted learning.

*H7*: Information system quality has a positive impact on perceived satisfaction of learners in GenAI-assisted learning.

*H8*: Interactive learning environment has a positive impact on perceived satisfaction of learners in GenAI-assisted learning.

*H9*: Perceived usefulness has a positive impact on perceived satisfaction of learners in GenAI-assisted learning.

### 2.10 Perceived self-regulation

Existing research has found a strong correlation between perceived satisfaction and perceived self-regulation (Kramarski and Gutman, 2006). For example, Ji et al. (2025) suggested that perceived satisfaction enhanced learners’ self-regulation, which in turn increases their intention to continue using GenAI. Zhou et al. (2024) also found that perceived usefulness was a crucial factor in promoting self-regulation in GenAI-assisted learning environments. Vighnarajah et al. (2009) also suggested that interactive e-learning environment could enhance learners’ self-regulation. Therefore, the following hypotheses were proposed:

*H10*: Perceived satisfaction has a positive impact on perceived self-regulation of learners in GenAI-assisted learning.

*H11*: Perceived usefulness has a positive impact on perceived self-regulation of learners in GenAI-assisted learning.

*H12*: Interactive learning environment has a positive impact on perceived self-regulation of learners in GenAI-assisted learning.

### 2.11 Behavioral intention

**Figure 2:**
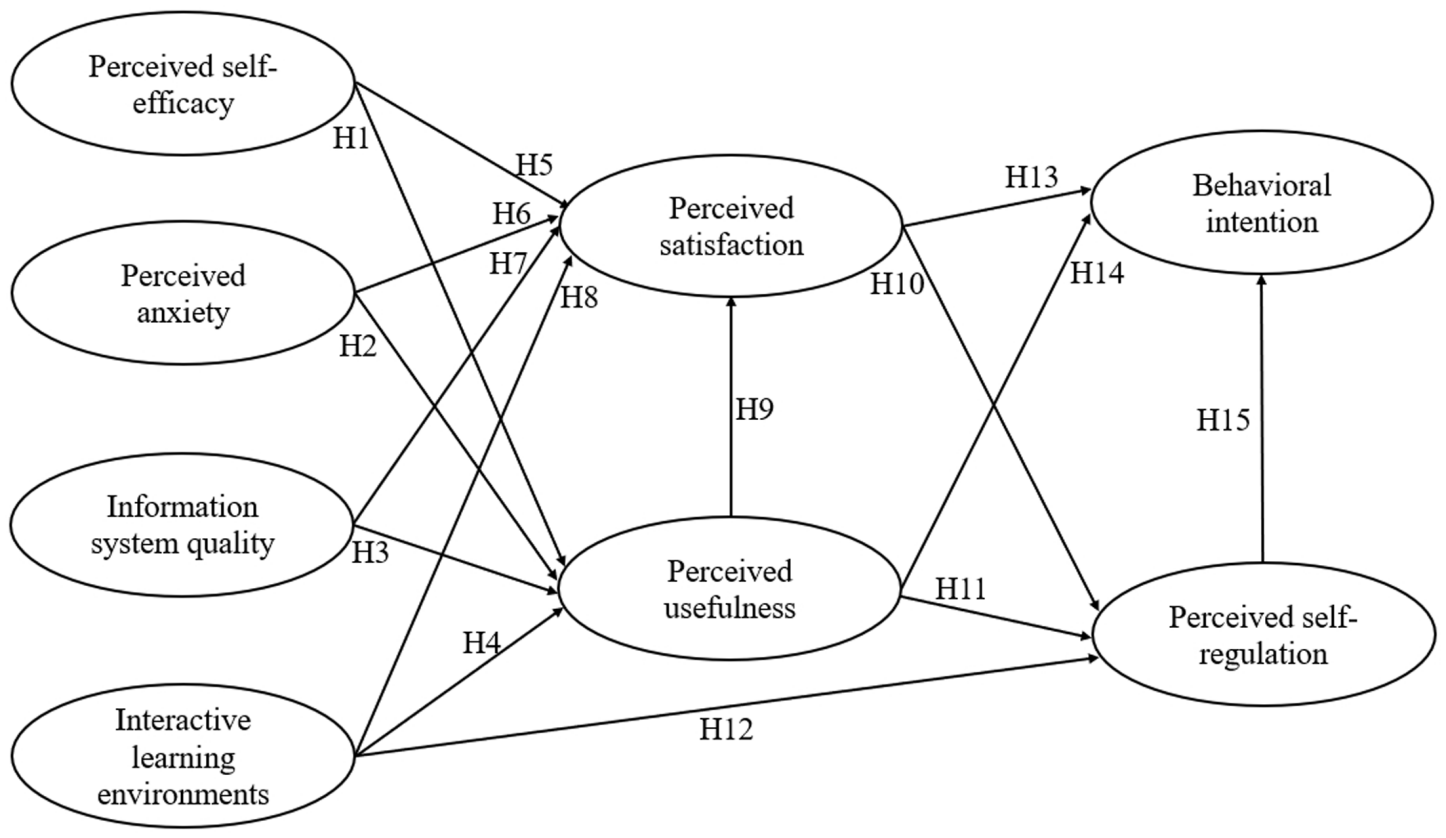
Research hypotheses.

This study defines behavioral intention as learner’s willingness to continue using GenAI (Cai et al., 2023). Existing studies have suggested that perceived satisfaction and usefulness could significantly influence learners’ behavioral intention in GenAI-assisted learning (Jung and Jo, 2025; Mohamed Eldakar et al., 2025). For example, Al-Sharafi et al. (2023) found that when students perceive AI chatbots as effective in assisting their learning tasks, their satisfaction also increases, enhancing their likelihood of continued use. Furthermore, Ma and Lee (2019) found that learners perceived usefulness and self-regulation were positively correlated with their behavioral intentions. Based on the above findings, we proposed the following hypotheses and research model (Figure 2).

*H13*: Perceived satisfaction has a positive impact on behavioral intention of learners in GenAI-assisted learning.

*H14*: Perceived usefulness has a positive impact on behavioral intention of learners in GenAI-assisted learning.

*H15*: Perceived self-regulation has a positive impact on behavioral intention of learners in GenAI-assisted learning.

## 3 Methods

This study employed SEM and qualitative interviews to examine the relationships among variables in GenAI-assisted learning. The methodological design was guided by Grant and Osanloo’s (2014) framework, which emphasizes that a theoretical framework serves as the blueprint of a research project. Their guidelines highlight the importance of (a) grounding a study in a clearly defined theoretical model, (b) ensuring alignment between the research questions, hypotheses, literature review, instruments, and data analysis procedures, and (c) providing a clear rationale for the selection of the theoretical foundation. Compared with other general methodological recommendations, such as Creswell and Creswell’s (2017) research design framework or Yin’s (2018) guidelines for case study research, Grant and Osanloo’s (2014) approach places a more explicit emphasis on integrating theory at every stage of the research process. This characteristic aligns with the current study, which requires a strong theoretical grounding to investigate the psychological and contextual factors affecting learners’ self-regulation in GenAI-assisted learning.

Following this guideline, the present study adopted Liaw and Huang’s (2013) three-tier model of self-regulation, as its theoretical foundation and expanded it into eight constructs to develop the research model. This alignment ensured theoretical coherence across the survey instrument, variable selection, and subsequent SEM analysis. To complement the quantitative findings, an open-ended interview question was appended to provide qualitative insights that enrich the interpretation of the results.

### 3.1 Instrument development

This study aimed to systematically test the hypotheses of the research model using a survey methodology. The questionnaire comprised three sections, with the first section collecting participants’ demographic information, including gender, age, educational level, major, and experience using GenAI. The second section comprised eight scales, each corresponding to a construct in the research model. The third section included an open-ended question designed to elicit participants’ views on the advantages, disadvantages, and suggestions for GenAI-assisted learning. This section was primarily based on the frameworks developed by Liaw (2008) and Liaw and Huang (2013), with relevant items revised to fit the context of this study. Therefore, the final research instrument consisted of 29 items measured on a seven-point Likert scale, ranging from 1 (strongly disagree) to 7 (strongly agree). Items numbered 1–3 evaluated perceived self-efficacy. Items 4–7 measured perceived anxiety. The information system quality was gauged through Items 8–11. Items 12–15 were formulated to assess the interactive learning environments, whereas Items 16–18 appraised their perceived satisfaction. Items 19–21 quantified the perceived usefulness. Items numbered 22–25 evaluated perceived self-regulation. Lastly, Items 26–29 determined the behavioral intention. The final version of the questionnaire is presented in Table A1.

Before the formal survey, a pilot survey was conducted to ensure the adaptability of the questionnaire items to the actual survey. However, the discriminate validity was not satisfactory between perceived self-efficacy and perceived satisfaction. Therefore, one of the items related to perceived self-efficacy was removed according to the results. Another item related to perceived usefulness was also removed due to its unacceptable factor loading.

To further improve the scientific validity of the scale, two experts with substantial experience in higher education and educational technology were invited to review the content of the questionnaire. The first expert is a professor specializing in technology integration in teacher education, with over 20 years of experience in developing and validating research instruments. The second expert is a senior researcher in psychometrics who has collaborated on several large-scale studies involving survey validation. These experts provided detailed feedback on the relevance, clarity, and validity of the questionnaire items, ensuring alignment with the study’s objectives and the theoretical framework. For example, in this study, the definition of “use” was based on the expected frequency of participants’ use of GenAI in current learning, rather than their future use. All items were carefully screened and adjusted for content validity. The items were then subjected to a back-translation validation process to ensure cross-cultural consistency and linguistic accuracy. Specifically, one researcher translated from English to Chinese and then another researcher translated the Chinese back to English.

### 3.2 Data collection

This study was conducted in Jiangsu Province, an economically developed coastal province in eastern Mainland China. Participants included undergraduate and graduate students majoring in math, etc. The Questionnaire Star website^1^ was used to pose the questionnaire. Counsellors from the school of mathematics at several universities in Jiangsu assisted us in sharing the link to the questionnaire with their university students via WeChat and Tencent QQ. While these recruitment methods helped us efficiently reach participants, it is important to acknowledge that these platforms may limit the diversity of the sample. WeChat and Tencent QQ are popular among younger, tech-savvy students, which may lead to an overrepresentation of certain demographic groups, such as more active and engaged students. Additionally, counselors from specific universities may have led to a concentration of participants from a limited number of institutions, potentially affecting the generalizability of the findings to students from other provinces or fields of study. At the beginning of the questionnaire, respondents were informed that they have the right to withdraw, confidentiality, anonymity and data protection. Participants voluntarily completed the survey and were entered into a small gift card lottery as appreciation for their participation. They were also informed about anonymity issues, explaining that the data would be used solely for research purposes. Ethical guidelines approved by the first author’s university academic committee were followed during data collection. Ethical approval was obtained under protocol number yzumath20240801 on August 1, 2024.

After data cleaning, the final analysis included data from 607 university students (e.g., prospective mathematics teachers) with experience using GenAI, aged 18–27. Participants were selected based on their use of GenAI, with popular tools being Baidu’s Ernie Bot and OpenAI’s ChatGPT. The demographic characteristics show that 37.4% of the respondents identity as males and 57.3% of the respondents are undergraduates. Additional details regarding the demographic characteristics of the respondents are presented in Table 1, with descriptive statistics.

**Table 1:** Demographic information about participants.

Item	Type	Frequency	Percentage (%)
Informed consent	Yes	607	100
	No	0	0
Gender identity	Male	227	37.4
	Female	380	62.6
	Non-binary	0	0
	Prefer not to say	0	0
Age	Under 19 years old	24	4.0
	20–22 years old	268	44.2
	23–25 years old	240	39.5
	Over 26 years old	75	12.4
Educational levels	Undergraduates	348	57.3
	Master’s students	222	36.6
	Ph.D. students	37	6.1
Major	Math	227	37.4
	Engineering	123	20.3
	Agriculture	46	7.6
	Medicine	39	6.4
	Arts and humanities	172	28.3

While we believe that these samples provide valuable insights, we also acknowledge that these recruitment methods introduce the possibility of selection bias. These samples may not fully represent the broader university student population, especially in terms of demographic diversity. Future work could benefit from expanding the recruitment methods to include a more varied range of platforms and institutions, which would enhance the representativeness of the sample and improve the external validity of the findings.

Once data collection was completed, we downloaded the data sets from the WJX platform for further analysis. The data were stored in SPSS 26 and saved in SAV format for normality testing.

### 3.3 Statistical approaches

Skewness and kurtosis indices were examined to assess the normality of each item, which is a prerequisite for SEM analysis. A dataset is generally considered normally distributed when the absolute skewness is below 3 and the absolute kurtosis is below 10 (Kline, 2023). The distribution of all scale items was examined using SPSS 26. Then, SPSS 26 and AMOS 28 were employed to evaluate the reliability and validity of the measurements and to test the proposed structural model. We also conducted a confirmatory factor analysis (CFA) using AMOS 28 to assess the measurement model. Prior to the CFA, the Kaiser–Meyer–Olkin (KMO) measure and Bartlett’s test of sphericity were examined to ensure sampling adequacy. The model included eight latent variables, and standardized factor loadings were evaluated to confirm construct validity. A factor loading threshold of 0.50 was adopted, and items with loadings below this threshold were removed to improve the reliability and validity of the measurement model.

Subsequently, Cronbach’s alpha (*α*), Average Variance Extracted (AVE), and Composite Reliability (CR) were calculated for each variable based on the retained items. Specifically, Cronbach’s α should exceed 0.80, the AVE should be greater than 0.50 and the CR should be above 0.70 (Hair et al., 2012). To assess the discriminant validity of the constructs, we employed the “Validity and Reliability Test” plugin in Amos, which incorporates both the Fornell–Larcker criterion and the Heterotrait–Monotrait (HTMT) ratio of correlations.

After confirming the validity and reliability of the constructs, we used Amos 28 to perform standardized model estimation via the maximum likelihood method. The analysis generated path coefficients (*β*) and significance levels (p), which were used to test the research hypotheses and evaluate the mediating effects. According to Baron and Kenny (1986), mediating effects can be classified into three types: full mediation, partial mediation, and suppressing mediation. In this study, mediation effects were tested using the bias-corrected and accelerated bootstrap method, with 5,000 resamples, as recommended in recent SEM practice (Hu et al., 2025; Tang et al., 2025). The explanatory power of the model for the outcome variables was evaluated by calculating the squared multiple correlations (R^2^). According to Hair and Alamer (2022), R^2^ values of 0.25, 0.50, and 0.75 are considered to represent weak, moderate, and substantial explanatory power, respectively.

Model fit indices in this study were evaluated based on widely accepted criteria, with the chi-square divided by degrees of freedom (χ^2^/df, CMIN/DF) expected to be ≤ 3.0 (Hayduk, 1987). The root mean square error of approximation (RMSEA) was expected to be ≤ 0.05 (Hair et al., 2017). The incremental fit index (IFI), Tucker–Lewis index (TLI), and comparative fit index (CFI) were required to be ≥ 0.90 (Bagozzi and Yi, 1988). The standardized root means square residual (SRMR) was considered acceptable if ≤ 0.08 (Hu and Bentler, 1999).

### 3.4 Qualitative analysis

To gain deeper insight into learners’ experiences with GenAI-assisted learning, we included one open-ended interview question at the end of the questionnaire: “Please tell us about the advantages, disadvantages and suggestions of GenAI.” This question was designed to elicit participants’ reflective opinions about GenAI, including perceived strengths, limitations, and potential improvements. The goal was to capture the subjective aspects of their experiences that might not be fully revealed through quantitative scales.

We conducted a thematic analysis on the open-ended responses to explore learners’ perceptions. All responses were reviewed and manually coded into three main themes—advantages, disadvantages, and suggestions—as aligned with the structure of the prompt. Each theme was further categorized into sub-themes based on lexical frequency and semantic similarity. Representative expressions were extracted for illustration.

The coding process was conducted using NVivo 12, and all codes were discussed and validated by two researchers to enhance reliability. The process involved iterative comparison and resolution of discrepancies through discussion.

## 4 Results

### 4.1 Measurement validity, reliability, and model fit indices

The results of the normality test are presented in Table 2. The skewness values ranged from −0.921 to 0.161, and the kurtosis values ranged from −1.014 to 1.890. These results indicate that the skewness and kurtosis of the dataset fall within acceptable limits, suggesting that all 29 items across the eight variables approximate a normal distribution.

**Table 2:** Descriptive statistics and normality tests.

Variable	Item	M	SD	Skewness	Kurtosis	Population M	Population SD
PSE	PSE01	5.45	1.098	−0.642	0.639	5.45	0.97
	PSE02	5.49	1.058	−0.921	1.890		
	PSE03	5.39	1.126	−0.568	0.332		
PA	PA01	4.11	1.633	0.066	−0.871	4.06	1.58
	PA02	4.17	1.706	0.035	−1.014		
	PA03	3.95	1.714	0.161	−1.007		
	PA04	4.01	1.744	0.005	−1.014		
ISQ	ISQ01	5.43	1.127	−0.773	1.018		
	ISQ02	5.47	1.175	−0.925	1.276	5.43	0.98
	ISQ03	5.40	1.134	−0.677	0.793		
	ISQ04	5.43	1.132	−0.659	0.874		
ILE	ILE01	5.23	1.189	−0.508	0.069	5.29	0.99
	ILE02	5.27	1.160	−0.546	0.352		
	ILE03	5.34	1.194	−0.686	0.758		
	ILE04	5.34	1.140	−0.321	−0.334		
PS	PS01	5.43	1.113	−0.713	1.036	5.44	0.97
	PS02	5.45	1.138	−0.610	0.435		
	PS03	5.43	1.137	−0.532	0.258		
PU	PU01	5.57	1.011	−0.515	0.221	5.48	0.91
	PU02	5.50	1.047	−0.587	0.640		
	PU03	5.37	1.134	−0.460	0.028		
PSR	PSR01	5.46	1.122	−0.560	0.212	5.46	0.92
	PSR02	5.46	1.055	−0.523	0.350		
	PSR03	5.49	1.114	−0.776	1.286		
	PSR04	5.41	1.103	−0.623	0.668		
BI	BI01	5.57	1.103	−0.927	1.847	5.46	0.97
	BI02	5.50	1.109	−0.854	1.336		
	BI03	5.33	1.176	−0.669	0.862		
	BI04	5.43	1.148	−0.830	1.217		

PSE, perceived self-efficacy; PA, perceived anxiety; ISQ, information system quality; ILE, interactive learning environments; PS, perceived satisfaction; PU, perceived usefulness, PSR, perceived self-regulation; BI, behavioral intention.

The proposed model demonstrated an excellent fit to the collected data. As shown in Table 3, the model fit indices were as follows: CMIN/DF = 1.865, RMSEA = 0.038, IFI = 0.973, TLI = 0.969, CFI = 0.973, and SRMR = 0.040. All six indices met the recommended thresholds, indicating a satisfactory model fit.

**Table 3:** Model fit indices.

Indices	Standards	Model indices values	Conclusion	Standard sources
CMIN/DF	<3	1.865	Excellent fit	Hayduk (1987)
RMSEA	<0.05	0.038	Excellent fit	Hair et al. (2017)
IFI	>0.9	0.973	Excellent fit	
TLI	>0.9	0.969	Excellent fit	
CFI	>0.9	0.973	Excellent fit	Bagozzi and Yi (1988)
SRMR	<0.08	0.040	Excellent fit	Hu and Bentler (1999)

As shown in Table 4, all items were satisfactorily loaded onto the eight factors corresponding to the proposed variables. Both the composite reliability (CR) and average variance extracted (AVE) values for all constructs exceeded the recommended thresholds. Additionally, the CFA results indicated a KMO value of 0.934, and Bartlett’s test of sphericity was significant (χ^2^ = 11,507.763, *p* < 0.001). Cronbach’s alpha values for all variables ranged from 0.811 to 0.946, indicating satisfactory internal consistency. Collectively, these results confirmed that the measurement model met the prerequisites for subsequent SEM analysis.

**Table 4:** Construct validity and convergent validity.

Variable	Item	Factor loading	Cronbach’s α	CR	AVE
PSE	PSE01	0.847***	0.865	0.865	0.682
	PSE02	0.819***			
	PSE03	0.811***			
PA	PA01	0.906***	0.946	0.946	0.814
	PA02	0.885***			
	PA03	0.918***			
	PA04	0.900***			
ISQ	ISQ01	0.822***	0.877	0.877	0.641
	ISQ02	0.789***			
	ISQ03	0.817***			
	ISQ04	0.774***			
ILE	ILE01	0.840***	0.870	0.871	0.628
	ILE02	0.791***			
	ILE03	0.785***			
	ILE04	0.752***			
PS	PS01	0.772***	0.826	0.827	0.615
	PS02	0.747***			
	PS03	0.832***			
PU	PU01	0.729***	0.811	0.808	0.583
	PU02	0.774***			
	PU03	0.787***			
PSR	PSR01	0.797***	0.862	0.862	0.610
	PSR02	0.737***			
	PSR03	0.792***			
	PSR04	0.797***			
BI	BI01	0.761***	0.879	0.880	0.648
	BI02	0.831***			
	BI03	0.841***			
	BI04	0.785***			

***p < 0.001. CR, composite reliability; AVE, average variance extracted.

Furthermore, The Fornell-Larcker criterion and the Heterotrait-Monotrait Ratio (HTMT) were employed to assess discriminant validity. As shown in Table 5, the square roots of the AVE for each construct exceeded the corresponding inter-construct correlations, indicating satisfactory discriminant validity (Fornell and Larcker, 1981). This result indicated that the study achieved satisfactory discriminant validity according to the Fornell–Larcker criterion. To further confirm these findings, the Heterotrait–Monotrait (HTMT) ratio of correlations was calculated, reflecting the average inter-item correlations across constructs compared with those within the same construct (Henseler et al., 2015). As shown in Table 6, all HTMT values were below the recommended threshold of 0.90, indicating acceptable discriminant validity (Hair et al., 2019).

**Table 5:** The Fornell-Larcker test results.

Constructs	Discriminate validity							
	PSR	BI	PU	PS	ISQ	ILE	PA	PSE
PSR	**0.781**							
BI	0.638	**0.805**						
PU	0.714	0.612	**0.764**					
PS	0.676	0.580	0.716	**0.784**				
ISQ	0.610	0.589	0.673	0.724	**0.801**			
ILE	0.591	0.574	0.681	0.606	0.579	**0.792**		
PA	−0.120	−0.118	−0.031	−0.038	−0.109	0.041	**0.902**	
PSE	0.510	0.522	0.514	0.506	0.473	0.510	−0.072	**0.826**

Diagonals in bold are the square roots of AVE and the lower triangle is the conformal Pearson correlation coefficient.

**Table 6:** The Heterotrait-Monotrait (HTMT) ratio testing results.

Constructs	PSE	PA	ILE	ISQ	PA	PU	BI	PSR
PSE								
PA	0.445							
ILE	0.413	0.04						
ISQ	0.432	0.098	0.511					
PS	0.436	0.042	0.518	0.628				
PU	0.456	0.031	0.576	0.571	0.594			
BI	0.441	0.11	0.507	0.517	0.503	0.525		
PSR	0.445	0.112	0.509	0.527	0.576	0.598	0.560	

### 4.2 Path analysis and research model power

The explanatory power of the hypothesized model is presented in Table 7. The R^2^ values for perceived usefulness, perceived satisfaction, perceived self-regulation, and behavioral intention were 0.625, 0.642, 0.590, and 0.496, respectively. The results indicate that the model demonstrates moderate to strong explanatory power for perceived usefulness, perceived satisfaction, and perceived self-regulation, while the relatively lower R^2^ value for behavioral intention. This suggests that the model accounts for only about half of the variance in behavioral intention, leaving a significant portion unexplained. The unexplained variance may be attributed to additional factors that were not considered in the current model. For example, social influence (e.g., peers, colleagues, or societal norms) may play a significant role in shaping students’ behavioral intentions, especially in technology adoption contexts (Zheng W. et al., 2024; Zheng Y. et al., 2024). Furthermore, platform trust could be another critical factor, as users’ trust in the GenAI platform may strongly affect their intention to use it (Wang et al., 2025). Also, perceived ease of use and previous experience with similar technologies could contribute to explaining the remaining variance in behavioral intention (Unal and Uzun, 2021). These factors, which were not included in this study, could provide a more comprehensive understanding of the factors influencing behavioral intention.

**Table 7:** Results of model path analysis.

No.	Hypothesis	Un Std.	S.E.	C.R.	*p*-value	Std. (β)	Support	R^2^
H1	PSE → PU	0.156	0.043	3.609	0.000	0.160	Yes	0.625
H2	PA → PU	−0.009	0.019	−0.456	0.648	−0.016	No	
H3	ISQ → PU	0.396	0.051	7.736	0.000	0.389	Yes	
H4	ILE → PU	0.402	0.055	7.373	0.000	0.386	Yes	
H5	PSE → PS	0.102	0.042	2.449	0.014	0.109	Yes	0.642
H6	PA → PS	0.007	0.018	0.394	0.694	0.013	No	
H7	ISQ → PS	0.408	0.056	7.287	0.000	0.416	Yes	
H8	ILE → PS	0.107	0.056	1.921	0.049	0.107	Yes	
H9	PU → PS	0.288	0.067	4.293	0.000	0.299	Yes	
H10	PS → PSR	0.324	0.065	4.99	0.000	0.311	Yes	0.590
H11	PU → PSR	0.427	0.074	5.799	0.000	0.426	Yes	
H12	ILE → PSR	0.116	0.058	2	0.045	0.111	Yes	
H13	PS → BI	0.182	0.064	2.826	0.005	0.186	Yes	0.496
H14	PU → BI	0.283	0.069	4.087	0.000	0.301	Yes	
H15	PSR → BI	0.276	0.062	4.476	0.000	0.294	Yes	

Overall, while the model shows a strong fit for several key variables, the relatively lower R^2^ for behavioral intention highlights the need for further exploration of other potential influences, such as social influence and platform trust, that might contribute to the formation of behavioral intention.

Additionally, the results of the path analysis showed that information system quality and interactive learning environments contributed more to perceived usefulness than perceived self-efficacy and perceived anxiety. Information system quality and perceived usefulness explained and predicted perceived satisfaction more significantly than perceived self-efficacy, perceived anxiety, and interactive learning environments. Second, perceived usefulness had a greater impact on perceived self-regulation than perceived satisfaction and interactive learning environments. Perceived usefulness and perceived self-regulation emerged as stronger predictors of behavioral intention than perceived satisfaction.

**Figure 3:**
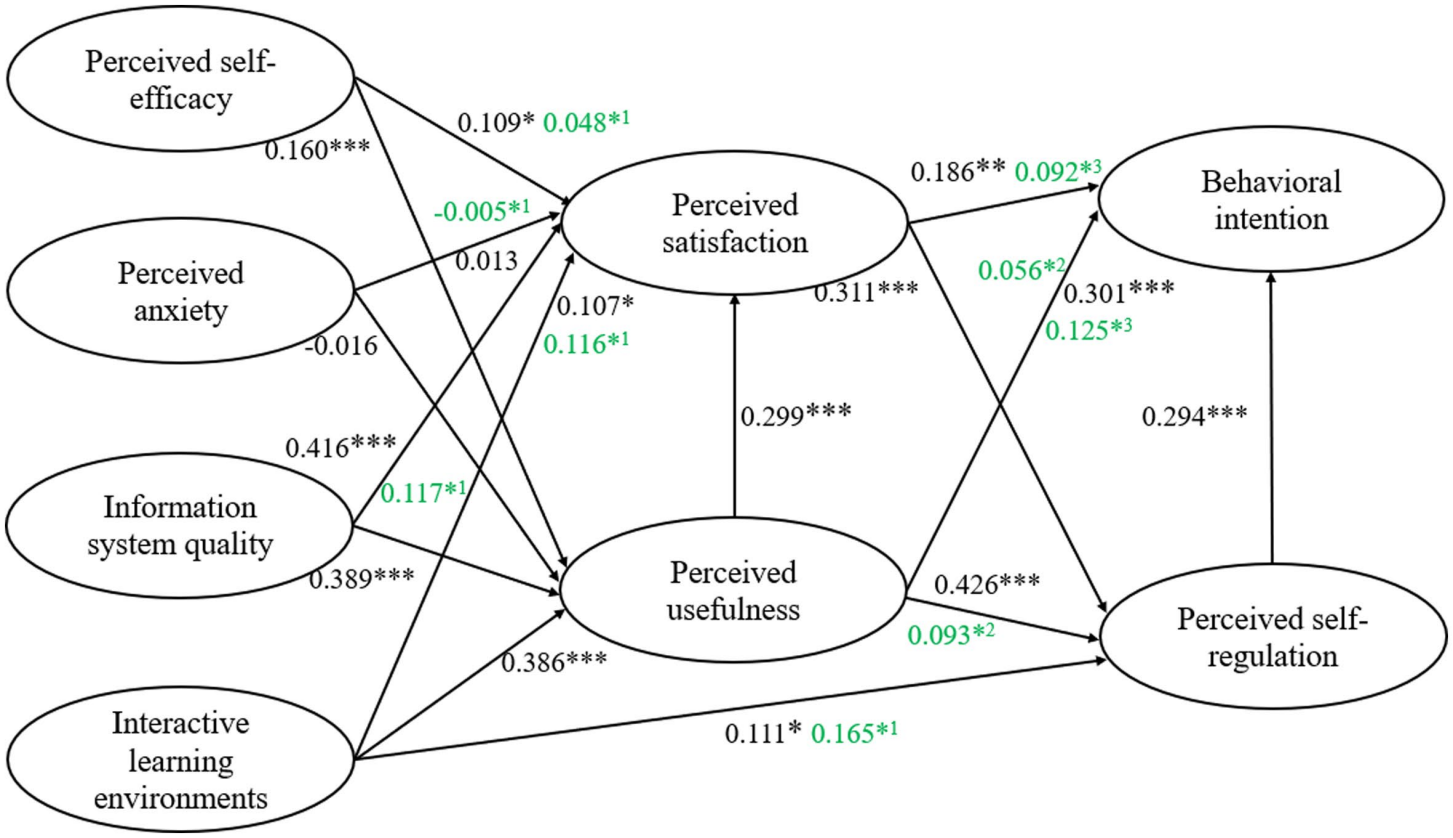
The results of hypotheses. The green numbers in the figure represent the indirect effect values. Superscript 1 indicates mediation through PU, superscript 2 indicates mediation through PS, and superscript 3 indicates mediation through PSR. ***p < 0.001, **p < 0.010, *p < 0.050.

Table 8 presents the mediating effects of perceived satisfaction, perceived usefulness and perceived self-regulation. The path of perceived anxiety on perceived satisfaction was not significant, as were the paths of perceived satisfaction and perceived usefulness on behavioral intention. All other mediating paths exhibited partial mediation effects. Figure 3 illustrates the final structural model.

**Table 8:** Mediating effects.

Path	Indirect effect	SE	p-value	95%CI
PSE → PU → PS	0.048	0.037	0.016	[0.008, 0.175]
PA → PU → PS	−0.005	0.014	0.595	[−0.040, 0.021]
ISQ → PU → PS	0.117	0.081	0.011	[0.025, 0.339]
ILE → PU → PS	0.116	0.071	0.011	[0.028, 0.307]
ILE → PU → PSR	0.165	0.095	0.015	[0.039, 0.406]
PU → PS → PSR	0.093	0.122	0.022	[0.017, 0.329]
PU → PS → BI	0.056	0.192	0.097	[−0.021, 0.173]
PU → PSR → BI	0.125	0.081	0.023	[0.026, 0.322]
PS → PSR → BI	0.092	0.067	0.028	[0.007, 0.283]

### 4.3 Qualitative results

Table 9 shows the key themes that emerged from the qualitative analysis. For advantages, learners frequently highlighted intelligent responses, fast and efficient interaction, and personalized learning support. Disadvantages focused on dependency on the tool, potential inaccuracies, and operational difficulties such as needing precise prompts. Suggestions included calls to enhance efficiency, improve personalization, strengthen accuracy, and promote broader awareness and usability of GenAI.

**Table 9:** Thematic analysis of learners’ perceptions in GenAI-assisted Learning.

Main theme	Sub-theme	Representative vocabulary	Representative quotes
Advantages	Intelligent performance	Intelligent response	“GenAI is like having a personal assistant who can always provide quick answers, especially when solving complex mathematical problems.”
	Efficiency and convenience	Convenient and fast, efficient	“It saves a lot of time, especially when working on assignments and mathematical proofs. I can quickly check solutions to problems.”
	Personalization and adaptability	Personalized	“I feel like GenAI provides personalized responses, but it still does not understand the context of my specific needs.”
Disadvantages	Over-reliance and reduced initiative	Overly dependent	“Sometimes I rely too much on GenAI, and it prevents me from thinking critically or attempting to solve math problems on my own.”
	Accuracy concerns	Inaccurate	“I’ve noticed that sometimes GenAI’s solutions to math problems are incorrect, especially with more complex calculus or algebra problems.” “I sometimes feel anxious when using GenAI because I’m not sure if the answers are correct, but it does not really affect my overall satisfaction.”
	Operational challenges	Demanding prompts, obtaining difficulties	“Sometimes the prompts I need to enter are so specific, and it takes a lot of effort to get the right response.”
Suggestions	System optimization	Improving efficiency and convenience	“If GenAI could better understand mathematical symbols and notation, it would be even more helpful for solving equations and proofs.”
	Enhanced personalization and creativity	Enhancing personalization and creativity	“It would be great if the system could be tailored more to my specific mathematical course needs, helping me think more creatively about problem-solving.”
	Accuracy improvement	Strengthening accuracy	“Improving the accuracy of responses would make the tool more reliable for academic work.”
	Promotion and accessibility	Widespread popularity	“GenAI is becoming really popular among students, and I think it will soon be an essential tool for all learners.”

## 5 Discussion

This study explored the effects of perceived anxiety on perceived usefulness and satisfaction in GenAI-assisted learning (H2 and H6). The results showed that perceived anxiety had no significant effect on these variables, which contradicts previous findings (Sallam et al., 2025; Zhu et al., 2024). One possible explanation is that reducing barriers to using GenAI may streamline the learning process, allowing learners to focus on self-regulation rather than being distracted by technology-related anxiety (Mohamed et al., 2025). Moreover, as GenAI tools (e.g., Ernie Bot and ChatGPT) have become widely adopted, learners have developed greater adaptability to their use, while the associated complexity and barriers have progressively diminished (Laun and Wolff, 2025). Therefore, perceived anxiety may no longer serve as a fundamental predictor of perceived usefulness and satisfaction. Over time, students have likely become more comfortable and confident in utilizing GenAI tools, reducing the emotional response to potential technology-related stressors. It is important to consider the possibility that the measurement limitations in this study may have contributed to these non-significant findings. The scales used to measure perceived anxiety, usefulness, and satisfaction may not have fully captured the complex relationship between these variables. Future studies could benefit from refining these measures or exploring alternative approaches to assess anxiety and its impact more effectively, particularly in contexts involving rapidly evolving technology like GenAI.

The study revealed that information system quality exerted a stronger influence on perceived usefulness and satisfaction than did the interactive learning environment and perceived self-efficacy (H1, 3, 4, 5, 7, and 8). This finding confirmed Zheng W. et al.’s (2024) and Zheng Y. et al. (2024) research. High-quality information systems provide learners with seamless user experience, reducing cognitive load and enabling them to concentrate on the learning process. In this study, the GenAI can deliver clear, structured responses that effectively support knowledge acquisition. This capability enables learners to quickly identify relevant information and complete learning tasks more efficiently. In this study, the overwhelming impact of information system quality might suggest that learners’ emotional and motivational responses to technology could be less emphasized in GenAI-assisted learning. However, this does not diminish the importance of psychological factors—in fact, they might become more significant in environments where learners are encouraged to explore and use these tools more independently. Emotional responses (e.g., satisfaction) related to learning challenges, the sense of control, and self-regulation could influence learners’ ability to fully engage with the tool and sustain its use over time. These findings imply that both technical and psychological factors must be integrated when constructing theoretical models for GenAI-assisted learning. A more holistic model would recognize the interaction between learner characteristics (e.g., self-efficacy, motivation), the technical quality of the tools (e.g., usability, personalization), and the emotional experiences during the learning process. Future work should focus on how these elements interact, as this could provide a more nuanced understanding of how GenAI tools affect learning outcomes, adoption, and sustained engagement.

Moreover, the interactive learning environment contributed more to perceived satisfaction than perceived self-efficacy, a finding consistent with the results of Jin et al. (2025b). A possible explanation is that university students develop foundational computer and AI interaction skills during their studies, leading to an almost negligible level of computer illiteracy in this population (Zhang and Wang, 2025). Consequently, learners tend to demonstrate high confidence in using GenAI tools, which diminishes the predictive effect of self-efficacy. Finally, the enhanced satisfaction observed in this study can be attributed to the provision of accurate information, concise content, and engaging interface design offered by the GenAI tools (Mun and Hwang, 2024). By presenting content in logically coherent and easily comprehensible components, these tools facilitate smoother learning experiences and enable the efficient completion of tasks. However, this finding is closely tied to the capabilities of Ernie Bot and ChatGPT and may not necessarily generalize all GenAI applications. Future work could extend this work by comparing a broader range of GenAI models to determine whether similar effects on perceived satisfaction and usefulness are observed across tools with varying capabilities. Examining how different GenAI functionalities shape learning outcomes would yield deeper insights to inform both tool selection and instructional design.

Additionally, this study found a significant and strong correlation between perceived usefulness and satisfaction (H9), aligning with previous findings (Kim et al., 2025). According to social exchange theory (Cook and Rice, 2003), learners who perceive the learning content provided by GenAI as highly beneficial are more likely to experience satisfaction with their learning experience. In other words, when learners perceive that GenAI enhances their learning performance and facilitates the effortless completion of tasks, their overall learning satisfaction tends to increase (Jung and Jo, 2025).

Perceived usefulness also played a significant partial mediating role in the relationships between perceived self-efficacy, information system quality, and interactive learning environment on perceived satisfaction. Among these paths, perceived usefulness served as the strongest mediator between information system quality and perceived satisfaction. The GenAI tools used in this study (e.g., Ernie Bot and ChatGPT) have gained widespread adoption among learners, mainly because they deliver rapid responses and high-quality learning content (Liu et al., 2025a). These capabilities help learners achieve their academic goals more effectively and enhance their overall satisfaction. In other words, higher system usability is positively associated with increased learner satisfaction (Almufarreh, 2024). Similarly, the perceived usefulness of GenAI tools partially mediated the effects of the interactive learning environment on perceived self-regulation. When learners perceive these tools as useful, they are more likely to engage actively in technology-mediated learning interactions (Bai and Wang, 2025). By improving the quality and frequency of teacher–learner communication, learners are more likely to receive timely feedback and adjust their learning strategies.

Perceived satisfaction, perceived usefulness, and the interactive learning environment were identified as crucial predictors of perceived self-regulation in GenAI-assisted learning (H10-12), which aligns with previous research (Ji et al., 2025). Specifically, the interactive learning environment plays a critical role in fostering self-regulation. For example, pedagogical strategies like group discussions and structured assignments help learners actively using GenAI, enhancing their self-regulatory skills (Zhou et al., 2024). Additionally, motivation serves as a crucial factor in GenAI-assisted learning. Intrinsic motivation—such as the satisfaction derived from using the tool—encourages deeper cognitive engagement, whereas extrinsic motivation—such as the perceived usefulness of GenAI—drives more practical, goal-oriented learning behaviors. Together, these motivational factors and an interactive environment help learners integrate GenAI effectively into their studies, promoting self-directed learning and alignment with individual learning goals.

Furthermore, perceived satisfaction was found to partially mediate the effect of perceived usefulness on self-regulation in GenAI-assisted learning with Ernie Bot and ChatGPT. As learners’ satisfaction with these GenAI tools increases, their motivation to engage in the learning process rises, which in turn enhances their self-regulation abilities (Ji et al., 2025). This positive feedback loop underscores the essential role of perceived usefulness in supporting self-regulated learning within these specific GenAI environments. Surprisingly, perceived satisfaction did not mediate the effect of perceived usefulness on behavioral intention. This may be because learners prioritize the usefulness of tools like ChatGPT over overall satisfaction. For example, even if learners feel dissatisfied with the subscription cost of ChatGPT, they are still likely to continue using it if they believe that it contributes to their academic achievement (Jo, 2024).

The perceived usefulness has a stronger impact on behavioral intention than perceived satisfaction and self-regulation (H13–H15), which aligns with previous studies (Mohamed Eldakar et al., 2025; Mohamed et al., 2025). This finding suggests that when learners perceive GenAI, such as Ernie Bot and ChatGPT, to be more useful, they are more likely to adopt them for learning in the future. However, it is important to consider the cultural and institutional contexts in which these findings were observed, particularly in the context of Mainland China. In China, the education system is largely exam-oriented, which places a significant emphasis on achieving high academic performance in standardized exams. This system may influence students’ perceptions of GenAI tools primarily in terms of their efficacy and utility for exam preparation, rather than the overall satisfaction with the tools themselves. As a result, students may prioritize the perceived usefulness of GenAI over other factors like satisfaction. This could explain why our findings show that perceived usefulness is a stronger predictor of behavioral intention than perceived satisfaction. In contrast, in Western educational contexts, where there is often greater emphasis on self-regulated learning and personalized learning tools, students may place more importance on the satisfaction they derive from using GenAI (Al-Sharafi et al., 2023). The institutional and cultural differences in educational priorities could explain why findings in Western studies (e.g., Sallam et al., 2025) emphasize perceived satisfaction as a stronger predictor of students’ behavioral intentions. Additionally, perceived self-regulation partially mediated the effect of perceived usefulness on behavioral intention. When learners recognize the usefulness of Ernie Bot and ChatGPT, they are likely to adjust their learning behaviors more actively to maximize the benefits of these tools. Zimmerman and Schunk (2001) also emphasized that self-regulation is a crucial mediator in technology acceptance, enhancing the effect of perceived usefulness on learners’ behavioral intentions. However, perceived self-regulation did not mediate the impact of perceived satisfaction on behavioral intentions. This may be because self-regulation primarily involves learners’ internal cognitive and behavioral processes (Liaw and Huang, 2013), which may not fully correspond to the external feedback and interactions provided by tools such as Ernie Bot and ChatGPT. This misalignment may weaken the link between learners’ self-regulatory behaviors and their intentions to continue using GenAI tools.

In this study, both quantitative and qualitative data were collected to explore learners’ perceptions of GenAI-assisted learning. The quantitative data, derived from the survey responses, provided numerical insights into constructs such as perceived usefulness, self-regulation, and behavioral intention. Meanwhile, the qualitative data, gathered from the open-ended responses, allowed us to capture participants’ subjective experiences and suggestions for improvement. To enhance the robustness of our findings, we conducted a triangulation process, where we compared and integrated both types of data. This process not only helped us cross-validate the findings but also enriched our interpretation of the results. For example, the quantitative data indicated a strong positive correlation between perceived usefulness and behavioral intention, which was supported by the qualitative feedback where many participants described how GenAI’s efficiency and convenience led them to use it more frequently for academic tasks. One participant stated, “It saves a lot of time, especially when working on assignments and mathematical proofs. I can quickly check solutions to problems.” This qualitative response aligns with the quantitative data showing that efficiency was a key predictor of behavioral intention in our model. Furthermore, the quantitative results revealed that perceived anxiety and perceived satisfaction had an insignificant path in the model, indicating that the level of anxiety felt by users did not significantly impact their satisfaction with GenAI. This result was further nuanced by qualitative data, where some participants expressed concerns about accuracy and ease of use, but did not directly associate these factors with overall satisfaction. As one participant mentioned, “I sometimes feel anxious when using GenAI because I’m not sure if the answers are correct, but it does not really affect my overall satisfaction.” This suggests that while anxiety may influence users’ perceptions of GenAI, it does not directly reduce their satisfaction with its use, possibly due to the tool’s overall convenience and efficiency. By integrating both types of data, we were able to validate and further explain the quantitative findings, demonstrating the consistency between the two datasets. This triangulation not only strengthens the validity of our conclusions but also provides a more nuanced understanding of the learners’ experiences with GenAI. The qualitative insights, particularly the suggestions for improving personalization and accuracy, help contextualize the quantitative results and offer actionable recommendations for future system optimization. In future studies, continuing to incorporate both qualitative and quantitative methods will allow for a deeper understanding of the complex factors influencing learners’ engagement with GenAI.

## 6 Limitations

This study has several limitations. First, the sample consisted of undergraduates and graduate students, with limited representation from other educational levels. Future research should include primary education and special education to capture a broader spectrum of learning experiences and enhance the generalizability of the findings. Second, the study used a cross-sectional design, which restricts the ability to capture the dynamic changes of learners’ self-regulation. Researchers should adopt longitudinal follow-up designs to examine how self-regulation in GenAI-assisted learning evolves over time. Third, this study included only an open-ended interview question in the questionnaire, which may not fully capture the participants’ subjective experiences in GenAI-assisted learning. Future work should consider incorporating in-depth interviews to obtain richer qualitative insights. Fourth, this study primarily reflects learners’ experiences with GenAI-assisted learning using Ernie Bot and ChatGPT, as these were the most popular tools among Chinese learners. Although the research instrument was designed to capture general perceptions of GenAI in learning, the findings may not fully reflect learners’ experiences with other GenAI tools. Consequently, the generalizability of the results to other GenAI tools may be limited. Future work should focus on specific GenAI applications or conduct comparative analyses across different platforms to gain a more comprehensive understanding of learners’ self-regulation in various GenAI-assisted learning environments. Fifth, while this study relied on a single open-ended interview question for qualitative input, we acknowledge that this design may limit the depth of the qualitative insights collected. The decision to use only one open-ended question was made to focus on a specific aspect of participants’ experiences with GenAI-assisted learning, ensuring that responses would be aligned with the research focus. However, we recognize that a broader set of open-ended questions could have allowed for a more nuanced exploration of participants’ perspectives on other relevant factors, such as their motivations, challenges, and perceived benefits. In future research, we plan to include a series of open-ended questions to gather more detailed and varied qualitative data. This would allow us to explore different dimensions of participants’ experiences and provide a richer understanding of the factors that influence engagement with GenAI-assisted learning. Additionally, this study relied on self-reported data, which may be susceptible to common method bias (CMB). Specifically, participants may have provided responses that align with social desirability or other personal biases, potentially inflating the relationships between the variables. Although the self-reported nature of the data is a common approach in educational research, the risk of CMB cannot be overlooked. To address this limitation, future work could incorporate techniques such as Harman’s single-factor test or the use of a variable marker to test for the presence of CMB. These methods would help ensure that the observed relationships are not unduly influenced by common method effects. Finally, one notable limitation is the lack of multi-group SEM (MGSEM) to examine potential differences in path relationships across subpopulations. This study focused on a general model without considering the possibility that different groups, such as those based on gender, education level, or other demographic factors, may exhibit distinct learning behaviors and responses to GenAI-assisted learning environments. MGSEM allows for a more nuanced understanding of heterogeneous learning behaviors, as it can explore whether path relationships vary across different subgroups. For instance, the effects of perceived usefulness or satisfaction on self-regulation or behavioral intention may differ for male and female participants, or for those at different levels of education. Therefore, future work could benefit from integrating MGSEM to explore these group differences and gain a more refined understanding of the diverse learning behaviors in GenAI-assisted learning environments.

## 7 Implications

From a theoretical perspective, this study offers empirical insights into learners’ self-regulation in GenAI-assisted learning, addressing a gap in existing literature and strengthening the theoretical foundation for applying GenAI in interdisciplinary learning. From a practical perspective, teachers are encouraged to integrate high-quality GenAI tools (e.g., ChatGPT in global contexts and Ernie Bot in China) into their teaching practices to enhance students’ learning experiences. By using these tools, educators can create personalized learning environments where students receive tailored feedback based on their individual needs. This integration enhances learners’ engagement by allowing them to interact with dynamic and creative content (e.g., AI-driven simulations, interactive lessons, and innovative assignments). Second, learners can actively engage in discussions with teachers to optimize their GenAI-assisted learning experiences (e.g., ChatGPT and Ernie Bot), adjusting timely their learning behaviors. Learners should also prioritize identifying valuable information and leveraging the high-quality resources provided by GenAI to develop a deeper understanding. Moreover, students can use GenAI as supportive tools for setting learning goals, managing their learning schedules, and monitoring progress, thereby enhancing their self-regulation. Third, operators should develop emotion-sensing systems to identify learners’ emotional states. Developers should prioritize user experience and system stability by creating learning systems that are user-friendly and accessible. Finally, developers can incorporate dynamic, interactive command designs into ChatGPT (e.g., layered prompts, visual navigation) to guide users step-by-step through operations. This approach not only reduces the learning threshold but also enhances the usability of ChatGPT.

## 8 Conclusion

This study expanded Liaw and Huang’s (2013) three-tier model of self-regulation into eight factors—perceived self-efficacy, perceived anxiety, interactive learning environments, information system quality, perceived satisfaction, perceived usefulness, perceived self-regulation, behavioral intention—to systematically examine the key determinants of learners’ self-regulation in GenAI-assisted learning. Findings revealed that the information system quality and interactive learning environments were stronger predictors of perceived usefulness than perceived self-efficacy. Information system quality was a more significant predictor of perceived satisfaction than both perceived self-efficacy and interactive learning environments, while perceived usefulness also played an effective role in predicting perceived satisfaction. Additionally, perceived usefulness was more effective in predicting the effects of perceived self-regulation than interaction learning environments and perceived satisfaction. It also outperformed both perceived satisfaction and perceived self-regulation in predicting behavioral intention. Furthermore, perceived usefulness partially mediated the effects of perceived self-efficacy, information system quality and interactive learning environments on perceived satisfaction. Perceived usefulness also partially mediated the effects of interactive learning environments on perceived self-regulation. Perceived satisfaction partially mediated the relationship between perceived usefulness and perceived self-regulation. Perceived self-regulation partially mediated the relationship between perceived usefulness and behavioral intention. This study found the factors influencing learners’ (e.g., prospective mathematics teachers) self-regulation in GenAI-assisted learning and further enriched the extended three-tier model of self-regulation.

## Data Availability

The raw data supporting the conclusions of this article will be made available by the authors, without undue reservation.

## Footnotes

1
http://www.wjx.cn
