# Supplementary material for: Perceived satisfaction, perceived usefulness, and interactive learning environments as predictors of university students’ self-regulation in the context of GenAI-assisted learning: an empirical study in mainland China
Source: Front Psychol. 2025 Dec 3;16:1599478. doi: 10.3389/fpsyg.2025.1599478 (PMC12722952; doi:10.3389/fpsyg.2025.1599478)
Supplement: Supplementary file 1 [file Supplementary_file_1.docx]

**Appendix**

**An investigation on the use of GenAI**

*Part 1: Informed consent and demographic information*

1. Would you like to participate in the study?

| □Yes | □NO |
| --- | --- |

2. Your gender is?

| □Male | □Female |
| --- | --- |

3. How old are you?

| □Below 19 years old | □20-22 years old | □23-25 years old | □26 years old and above |
| --- | --- | --- | --- |

4. Your grade is?

| □Middle school students | □Undergraduates | □Master’s students | □Doctoral candidates |
| --- | --- | --- | --- |

5. Your major belongs to?

| □Math | □Engineering | □Agriculture | □Medicine | □Arts and humanities |
| --- | --- | --- | --- | --- |

6. How often do you “use” GenAI?

| □Never | □Seldom | □Sometimes | □Often | □Always |
| --- | --- | --- | --- | --- |

*Part 2*

**Table A1**

*Eight subscales*

| Indicator | Questionnaire items | Questionnaire contents |
| --- | --- | --- |
| Perceived self-efficacy | PSE01 | When using GenAI to assist in completing a social practice report, do you have confidence in their effectiveness? |
|  | PSE 02 | When using GenAI to answer mathematical questions, do you feel fully confident in your ability to operate these tools effectively? |
|  | PSE03 | When using GenAI to obtain article readings, do you have confidence in the quality of the content it provides? |
| Perceived anxiety | PA01 | Using GenAI to write articles makes me feel awful. |
|  | PA02 | When using GenAI to solve complex math problems, I feel anxious and uneasy. |
|  | PA03 | Using GenAI to write class summaries makes me feel uncomfortable. |
|  | PA04 | I feel nervous when using GenAI to assist with course assignments. |
| Information system quality | ISQ01 | I am very satisfied with the capabilities of GenAI in summarizing text. |
|  | ISQ02 | I am satisfied with the response speed of GenAI during the text refinement process. |
|  | ISQ03 | I am satisfied with the writing learning content provided by GenAI. |
|  | ISQ04 | I am satisfied with the interactive performance of GenAI. |
| Interactive learning environments | ILE01 | I believe that using GenAI to assist with learning can assist teacher–learner interaction. |
|  | ILE02 | I believe that using GenAI to assist with learning can foster interaction among learners. |
|  | ILE03 | My classmates want to share their learning experiences using GenAI in writing. |
|  | ILE04 | The interactive features of GenAI in problem-solving can improve academic performance. |
| Perceived satisfaction | PS01 | I am satisfied with the writing learning resources provided by GenAI. |
|  | PS02 | I am satisfied with the content organization and presentation style of GenAI in editing. |
|  | PS03 | I am satisfied with the interactive features of GenAI in language practice. |
| Perceived usefulness | PU01 | I believe that GenAI is a useful tool for language learning. |
|  | PU02 | I believe that using GenAI to assist with learning can help me achieve my learning goals. |
|  | PU03 | I believe that using GenAI to assist with learning can enhance my motivation to learn. |
| Perceived self-regulation | PSR01 | GenAI is a tool that supports self-regulated learning. |
|  | PSR02 | GenAI is a learning tool that is easy to personalize and can meet my individual learning needs. |
|  | PSR03 | I can independently adjust the learning content in GenAI to suit my learning needs. |
|  | PSR04 | The content in the writing learning process assisted by GenAI is easy to adjust. |
| Behavioral intention | BI01 | I plan to use GenAI to assist with my learning in the future. |
|  | BI02 | I plan to use the learning content provided by GenAI to assist my studies. |
|  | BI03 | I plan to use GenAI to enhance my learning intentions. |
|  | BI04 | I plan to use GenAI as a tool for self-directed learning. |

*Part 3: an open-ended interview question*

Please tell us about the advantages, disadvantages and suggestions of GenAI.
